# Fate of cerium dioxide nanoparticles in endothelial cells: exocytosis

**DOI:** 10.1007/s11051-015-3007-4

**Published:** 2015-05-05

**Authors:** Claudia Strobel, Hartmut Oehring, Rudolf Herrmann, Martin Förster, Armin Reller, Ingrid Hilger

**Affiliations:** Department of Experimental Radiology, Institute of Diagnostic and Interventional Radiology, Jena University Hospital – Friedrich Schiller University Jena, Erlanger Allee 101, 07747 Jena, Germany; Institute of Anatomy II, Jena University Hospital – Friedrich Schiller University Jena, Teichgraben 7, 07743 Jena, Germany; Department of Physics, University of Augsburg, Universitaetsstraße 1, 86159 Augsburg, Germany; Department of Internal Medicine I, Division of Pulmonary Medicine and Allergy/Immunology, Jena University Hospital – Friedrich Schiller University Jena, Erlanger Allee 101, 07747 Jena, Germany

**Keywords:** Cerium dioxide, Endothelial cells, Exocytosis, Exocytosis inhibitor, Nanoparticle, Health effects

## Abstract

Although cytotoxicity and endocytosis of nanoparticles have been the subject of numerous studies, investigations regarding exocytosis as an important mechanism to reduce intracellular nanoparticle accumulation are rather rare and there is a distinct lack of knowledge. The current study investigated the behavior of human microvascular endothelial cells to exocytose cerium dioxide (CeO_2_) nanoparticles (18.8 nm) by utilization of specific inhibitors [brefeldin A; nocodazole; methyl-β-cyclodextrin (MβcD)] and different analytical methods (flow cytometry, transmission electron microscopy, inductively coupled plasma mass spectrometry). Overall, it was found that endothelial cells were able to release CeO_2_ nanoparticles via exocytosis after the migration of nanoparticle containing endosomes toward the plasma membrane. The exocytosis process occurred mainly by fusion of vesicular membranes with plasma membrane resulting in the discharge of vesicular content to extracellular environment. Nevertheless, it seems to be likely that nanoparticles present in the cytosol could leave the cells in a direct manner. MβcD treatment led to the strongest inhibition of the nanoparticle exocytosis indicating a significant role of the plasma membrane cholesterol content in the exocytosis process. Brefeldin A (inhibitor of Golgi-to-cell-surface-transport) caused a higher inhibitory effect on exocytosis than nocodazole (inhibitor of microtubules). Thus, the transfer from distal Golgi compartments to the cell surface influenced the exocytosis process of the CeO_2_ nanoparticles more than the microtubule-associated transport. In conclusion, endothelial cells, which came in contact with nanoparticles, e.g., after intravenously applied nano-based drugs, can regulate their intracellular nanoparticle amount, which is necessary to avoid adverse nanoparticle effects on cells.

## Introduction

The impact of nanotechnology in various branches of industry and in medicine has increased in the last years, which is reflected by nanoparticles’ use, for example, in certain products of the food sector (Chaudhry et al. 2008), or for prospective medical applications [e.g., for optical imaging (Jiang et al. 2010), for cancer therapy (Hilger 2013; Johannsen et al. 2005), or for drug delivery (Cho et al. 2008)], as contrast agents (Hahn et al. 2011), in cosmetics like sun protection agents (Strobel et al. 2014a) etc. Therefore, humans are increasingly faced with nanoparticles in daily life. The loading of cells with nanoparticles plays an important role for nanoparticles’ biocompatibility. In this context, there are many studies dealing with nanoparticles’ uptake in cells by endocytosis processes (Chithrani et al. 2006; Kim et al. 2006; Lesniak et al. 2012; Ma et al. 2013; Meng et al. 2011; Treuel et al. 2013). Such studies revealed that nanoparticles’ endocytosis is a concentration-, time- and energy-dependent process (Panyam and Labhasetwar 2003) and that it is mediated by clathrin, caveolae, and other mechanisms (Canton and Battaglia 2012). Moreover, it was shown that endocytosis of nanoparticles is dependent on cell type and on nanoparticles’ properties, like size, shape, and surface chemistry [(Canton and Battaglia 2012), and reviewed in (Oh and Park 2014)].

However, cell loading with nanoparticles is not only dependent on uptake, but also on time of intracellular retention and therefore on the behavior of cells to excrete internalized nanoparticles. A comprehensive understanding of exocytosis is of relevance for nanotoxicity assessments and for toxicity categorization of nanomaterials. Nevertheless until now exocytosis of nanoparticles has been the subject of only few studies [reviewed in (Oh and Park 2014)]. Examples are exocytosis of silica (Chu et al. 2011; Hu et al. 2011), gold (Bartczak et al. 2012; Chithrani and Chan 2007; Wang et al. 2011), or of polymer nanoparticles (Dombu et al. 2010; He et al. 2013a, b; Panyam and Labhasetwar 2003) in several tumor and non-tumor cell lines. Based on theses studies, it seems that exocytosis is a dynamic and energy-dependent process (Panyam and Labhasetwar 2003) like endocytosis. It is dependent on cell type (Chithrani and Chan 2007; Chu et al. 2011; Wang et al. 2011), nanoparticle amount in supernatants (Chu et al. 2011), and the nanoparticles’ properties like size (Chithrani and Chan 2007; Hu et al. 2011), shape (Chithrani and Chan 2007), and functionalization (Bartczak et al. 2012). Some studies demonstrated an involvement of cell membrane cholesterol (Dombu et al. 2010) and of intracellular membrane transport in exocytosis processes (He et al. 2013a, b).

Interestingly, cerium dioxide (CeO_2_) nanoparticles have been suggested to be included in cosmetics as UV filters and ROS scavengers (Boutard et al. 2013; Truffault et al. 2012; Yabe and Sato 2003) or in drugs for the treatment of medical disorders (Chigurupati et al. 2013; Karakoti et al. 2008; Niu et al. 2007; Schubert et al. 2006; Silva 2006). Therefore, a direct exposure of CeO_2_ nanoparticles with endothelial cells will occur, particularly if CeO_2_ nanoparticles will be used in intravenously applied medications. Moreover, CeO_2_ nanoparticles are present in the air due to their utilization in automobile catalytic converters (Zheng et al. 2005) and as automotive fuel additives (Jung et al. 2005; Park et al. 2008). It was shown that CeO_2_ nanoparticles were taken up by endothelial cells and were located perinuclearly (Strobel et al. 2014b), but it is unclear whether they can be exocytosed from cells.

Therefore, the objective of the present study was to determine the behavior of endothelial cells to exocytose CeO_2_ nanoparticles and we asked the following questions: (1) if nanoparticle intracellular accumulation decreases with increasing time after exposure, (2) which amounts of nanoparticles are detectable in the cell supernatants as a general measure of exocytosis with increasing time after exposure, (3) if nanoparticles are re-arranged within cells after nanoparticle exposure, and (4) which cellular components are involved in the exocytosis processes.

## Materials and methods

### Synthesis of the nanoparticles

Reagents and solvents of synthesis were obtained from Merck KGaA and Sigma-Aldrich if not otherwise specified. Ethanol (absolute for analysis) was used throughout the study.

CeO_2_ nanoparticles were synthesized using the method of Chen and Chang (Chen and Chang 2004, 2005). A solution of cerium(III) nitrate hexahydrate (3 mmol, 1.30 g) in 30 ml of water was stirred at 85 °C (oil bath temperature) in a round-bottom flask, and 1.5 ml of aqueous ammonia (25 %) was added. Stirring was continued for 3.5 h while allowing contact of the solution with air. After cooling to room temperature and stirring for 15 h, the suspension was centrifuged at 6,700 g for 15 min, and the precipitated nanoparticles were purified by redispersion in water (24 ml) and centrifugation (repeated 3 times), followed by redispersion in ethanol (24 ml) and centrifugation (repeated 3 times). They were stored in ethanol (9 ml). The yield was 340 mg (51 %) of CeO_2_ nanoparticles. Before their utilization in experiments, the nanoparticles were redispersed in sterile Millipore water (centrifugation and redispersion in 1.0 ml Millipore water; repeated 4 times), were vortexed and treated with ultrasound (10 min; ultrasound bath Bandelin Sonorex RK 52 H, Bandelin electronic GmbH & Co. KG, Germany; HF-power: 60 W_eff_).

Unlabeled nanoparticles were analyzed via transmission electron microscopy (TEM) and inductively coupled plasma mass spectrometry (ICP-MS). The flow cytometry analysis was carried out with ATTO 647 N-labeled nanoparticles.

**Fig. 1:**
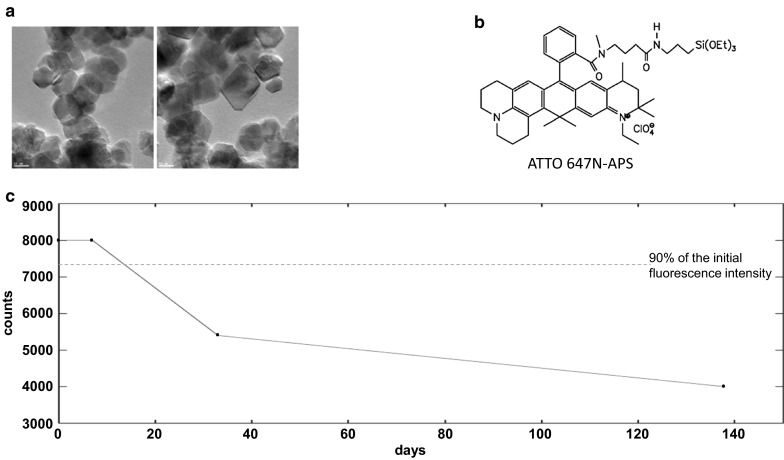
Nanoparticles’ features. **a** TEM pictures of the CeO_2_ nanoparticles show the varying shapes. **b** Structure of the reactive dye species ATTO 647 N-APS used for labeling. **c** Stability test of the ATTO 647 N-APS label in endothelial cell culture medium (PAA Laboratories, Pasching, Austria) revealed the stability of the dye for at least 2 weeks, since 90–95 % of the initial fluorescence intensity of the ATTO 647 N-APS label was present after this period

The labeling reagent ATTO 647 N-APS was prepared from commercial ATTO 647 N NHS ester (Sigma-Aldrich) by reaction with (3-aminopropyl)-triethoxysilane (APS). Thus, 0.9 µmol (0.75 mg) of the NHS ester was dissolved in 70 µl of dimethylformamide and stirred with 2.0 ml of an APS solution (1 mM) in ethanol for 2 h at room temperature. Completeness of the reaction (formation of the labeling reagent ATTO 647 N-APS) was checked by thin layer chromatography (silica, chloroform/ethanol 2:1, movement along the plate: retardation factor (*R*_f_) NHS ester 0.7, *R*_f_ ATTO 647 N-APS 0.8). The solution was diluted with 0.9 ml of ethanol to obtain a 0.32 mM stock solution of the reactive dye species (structure see Fig. 1b), which can be stored at 5 °C for at least 2 months (data not shown).

The stability of the label at room temperature in endothelial cell culture medium was checked by monitoring the fluorescence spectra on storage in the dark. To 3.0 ml of the medium, 20 µl of the ATTO 647 N-APS stock solution was added, mixed by shaking and then measured directly (excitation 640 nm, maximum emission at 660 nm; F900 luminescence spectrometer; Edinburgh Analytical Instruments, UK) at the time points shown in Fig. 1c.

The CeO_2_ nanoparticles (100 mg) were dispersed in ethanol (3 ml) in a 4-ml glass vial with screw cap and Teflon gasket (Wheaton). After addition of 150 µl of the ATTO 647 N-APS stock solution, the tightly closed vial was stirred at 120 °C (oil bath temperature) for 3 h. The label was covalently bonded to the nanoparticle surface by a transesterification reaction of the triethoxysilyl group with hydroxy groups on the CeO_2_ surface. The particles were isolated by centrifugation (11,000 g; 15 min). Unreacted ATTO 647 N-APS was removed by redispersion/centrifugation with ethanol (6 ml, 5 repetitions). The particles were stored in ethanol. Fluorescence spectra in ethanol dispersion showed appreciable labeling.

### Nanoparticle characterization

To characterize the used nanoparticles, the shape and size were determined by TEM (JEM 2100 F instrument; Jeol, Tokyo, Japan). The samples were prepared by spreading ethanol dispersions of the nanoparticles on a carbon film supported on a 200-mesh copper grid (Plano GmbH) and drying in air. TEM pictures of the nanoparticles were analyzed with the program ImageJ (http://rsb.info.nih.gov/ij/). The diameter of the circumscribed sphere for all nanoparticles was measured to obtain their average size due to their different morphologies.

The hydrodynamic diameters and the ζ-potentials of CeO_2_ nanoparticles (50 µg/ml) in water and cell culture medium (Gibco^®^ MCDB 131 medium (Life Technologies GmbH, Germany), 10 % (v/v) fetal bovine serum (FBS; Life Technologies GmbH, Germany), 1 % (v/v) GlutaMAX™ I 100X (Life Technologies GmbH, Germany), 1 µg/ml hydrocortisone (Sigma-Aldrich Chemie GmbH, Germany), 10 ng/ml epidermal growth factor (Life Technologies GmbH, Germany)) were measured using a zetasizer apparatus (Nano ZS Malvern Instruments, UK).

The cytotoxicity of similar CeO_2_ nanoparticles was investigated in a previous investigation (Strobel et al. 2014b) showing that the used nanoparticle concentrations of the present study did not affect the cells adversely.

### Cell cultures

Immortalized human microvascular endothelial cells (HMEC-1; Centers for Disease Control and Prevention, USA) were grown in cell culture medium (Gibco^®^ MCDB 131 medium (Life Technologies GmbH, Germany), 10 % (v/v) FBS (Life Technologies GmbH, Germany), 1 % (v/v) GlutaMAX™ I 100X (Life Technologies GmbH, Germany), 1 µg/ml hydrocortisone (Sigma-Aldrich Chemie GmbH, Germany), 10 ng/ml epidermal growth factor (Life Technologies GmbH, Germany)) in a humidified incubator at 37 °C in a 5 % CO_2_ atmosphere by changing the cell culture medium every 2–3 days. Cell cultures were subcultivated until reaching 70–85 % confluence using GIBCO^®^ trypsin (Life Technologies GmbH, Germany) and were free of mycoplasma as it was regularly tested by PCR.

### Investigation of exocytosis via flow cytometry

HMEC-1 were incubated with 1 µg/ml CeO_2_-ATTO 647 N for 24 h. Then the cells were washed with Hank`s BSS (Biochrom AG, Germany) and fresh nanoparticle-free medium was added. After several time points, the cells were measured via flow cytometry [10,000 cells; FACS Calibur (Becton–Dickinson GmbH, Germany); 635 nm laser; filter: FI4 661/16; CellQuest Pro™ software (Becton–Dickinson GmbH, Germany)]. For the long time follow-up (up to 240-h follow-up time), the median fluorescence intensity (MFI) of the appropriate untreated cell population was subtracted from the MFI of nanoparticle-exposed cells and the ratio of MFI of follow-up (“48 h”–“240 h”) to initial value (“0 h” follow-up; cells which were exposed to CeO_2_ nanoparticles for 24 h) was calculated.

To study the role of cellular constituents in exocytosis of nanoparticles, different inhibitors were used which affected different cellular structures/components. After the 24 h nanoparticle internalization, the cells were washed and exposed either to fresh nanoparticle-free medium, to Brefeldin A Ready Made Solution (0.1 µg/ml for 24 h; Sigma-Aldrich Chemie GmbH, Germany), to InSolution™ Nocodazole (10 µg/ml for 24 h; Merck KGaA, Germany), or to methyl-β-cyclodextrin (MβcD) (10 mM for 2 or 1 h, follow-up: 22 or 23 h, respectively; Sigma-Aldrich Chemie GmbH, Germany). 24 h after addition of fresh medium or inhibitor, 10,000 cells were analyzed via flow cytometry (FACS Calibur (Becton–Dickinson GmbH, Germany); 635 nm laser; filter: FI4 661/16; CellQuest Pro™ software (Becton–Dickinson GmbH, Germany)). The exocytosis rate of each sample was calculated using the MFI: The MFI of the appropriate control cell population was subtracted from the MFI of corresponding nanoparticle exposed cells. The percentile ratio of MFI of follow-up (“24 h” follow-up after washing and medium exchange) to initial value (“0 h” follow-up; cells which were exposed to CeO_2_ nanoparticles for 24 h) was calculated and subtracted from 100 %, resulting in the exocytosis rate.

### Investigation of exocytosis via TEM

HMEC-1 were seeded in 12-well plates and incubated with 10 µg/ml of CeO_2_ nanoparticles for 24 h. To investigate the exocytosis of the nanoparticles, 24 h after nanoparticle treatment the cells were washed and a cell culture medium exchange followed to remove the non-internalized nanoparticles. To inhibit the exocytosis of certain samples, cells were treated either with Brefeldin A Ready Made Solution (0.1 µg/ml for 24 h; Sigma-Aldrich Chemie GmbH, Germany), with InSolution™ Nocodazole (10 µg/ml for 24 h; Merck KGaA, Germany), or with MβcD (10 mM for 2 h, follow-up: 22 h; Sigma-Aldrich Chemie GmbH, Germany) in cell culture medium. 24 h after medium exchange, the cells were washed with Hank`s BSS (Biochrom AG, Germany) and fixed for 30 min at 20 °C with 2 % glutaraldehyde solution in 0.1 M cacodylate buffer (pH 7.4, 5 % sucrose). After repeated rinsing in 0.1 M cacodylate buffer (pH 7.4, 6.8 % sucrose), specimen was postfixed with a freshly prepared mixture of 2 % osmiumtetroxide (in distilled water) and 3 % potassium ferrocyanide (0.2 M cacodylate, pH 7.4) for 2 h at 4 °C followed by thorough washing in 0.1 M cacodylate buffer (pH 7.4) until the solution remained clear. Tissue sample was dehydrated in graded ethanol series and embedded in Epon 812 (FERAK, Berlin, Germany) via acetonitrile as inter medium. Samples were polymerized at 60 °C for 7 days. Ultrathin sections prepared with low-angle diamond knives were mounted on formvar-coated copper rhodium grids and stained with 1 % uranylacetate (in methanol) and freshly prepared lead citrate (25 mg/10 ml distilled water). Sections were examined by an EM 902A (ZEISS, Oberkochen, Germany) operating with an accelerating voltage of 80 kV.

### Investigation of exocytosis via ICP-MS

HMEC-1 were treated with 100 µg/ml CeO_2_ nanoparticles to be above the detection limit of the method. After 24 h of nanoparticle exposure, the cells were washed and nanoparticle-free cell culture medium was added to the cells either with or without MβcD (10 mM for 2 h, follow-up: 22 h; Sigma-Aldrich Chemie GmbH, Germany). After appropriate incubation time, the cell culture supernatant was collected. 7 ml HNO_3_ (HNO_3_ 65 %; Merck, cleaned by subboiling distillation) was added to 1 ml of each cell culture supernatant and a microwave-assisted digestion (Mars 5Xpress, CEM) followed. The cell culture supernatant samples were filled up to a final volume of 25 ml with deionized water (GenPure UV-TOC, Fisher Scientific). The concentration of cerium (Ce) in the appropriate digestion solution was determined via ICP-MS (XSeriesII, Thermo Fisher Scientific). For each sample, three measurements were done.

### Statistical analysis

During data analysis, the mean values and the standard deviations were calculated. Statistical data evaluation was carried out via ANOVA and post hoc Bonferroni test using IBM SPSS Statistics (version 22.0, Inc, IBM Company, USA). Data were stated as statistically significant if *P* ≤ 0.05.

## Results

### CeO_2_ nanoparticle characterization

The morphology of the used nanoparticles varied from octahedral to spherical (Fig. 1a; Table 1) with an average size of 18.8 ± 4.5 nm (Table 1). The degree of clustering varied over a large range, but it could not be quantified from TEM pictures. Dynamic light scattering (DLS) measurements suggested a strong clustering behavior of nanoparticles in the presence of cell culture medium (Table 1). The ζ-potential of nanoparticles changed from positive to negative when the nanoparticles were suspended in FBS-containing cell culture medium (10 %) indicating the occurrence of protein adsorption on the nanoparticles’ surface (Table 1). Unlabeled and labeled nanoparticles revealed similar properties (Table 1). Therefore, nanoparticle labeling should have no distinct effect on their uptake and exocytosis by the target cells.

**Table 1:** Characterization of the used CeO_2_ nanoparticles regarding shape, size, and ζ-potential

	With ATTO dye^d^	Without ATTO dye^e^
Shape	Octahedral/spheres	Octahedral/spheres
Size (nm)^a^	18.8 ± 4.5	18.8 ± 4.5
Size in H_2_O (nm)^b^	65 ± 1	79 ± 1
Size in cell culture medium (nm) shortly after preparation^b,c^	309 ± 9	369 ± 16
Size in cell culture medium (nm) after 3 h incubation^b,c^	307 ± 1	352 ± 6
ζ-potential in H_2_O (mV)	18.7 ± 0.5	18.4 ± 1.0
ζ-potential in cell culture medium (mV)^c^	−24.1 ± 0.3	−23.8 ± 0.6

^a^By TEM

^b^By DLS (polydispersity index <0.5)

^c^Cell culture medium supplemented with 10 % FBS

^d^Nanoparticles which were labeled with the dye ATTO 647 N-APS were used in flow cytometry analysis

^e^Unlabeled counterparts were used for TEM and ICP-MS analysis

The labeled nanoparticles were stable for at least two weeks, since 90–95 % of the initial fluorescence intensity of the ATTO 647 N-APS label was present after this period of time, as the stability test in cell culture medium showed (Fig. 1c).

### Exocytosis of nanoparticles

#### Decrease of intracellular nanoparticle accumulation and partial re-uptake of exocytosed nanoparticles with increasing time after exposure

**Fig. 2:**
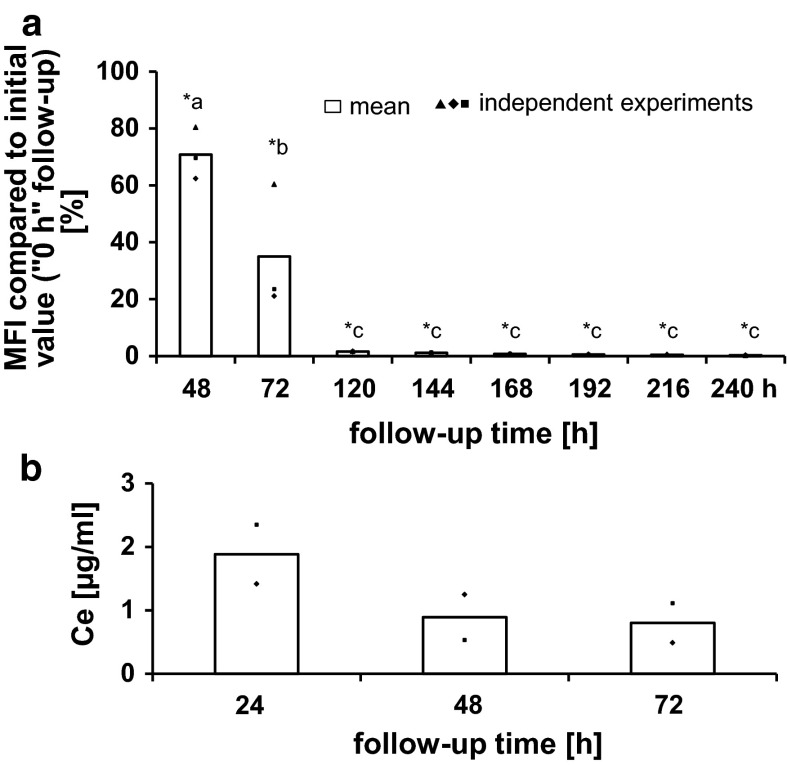
Decrease of intracellular nanoparticle accumulation and partial re-uptake of exocytosed nanoparticles with increasing time after exposure. **a** With increasing follow-up time after nanoparticle exposure a continuous decrease of the intracellular fluorescence intensity was observed. This indicates a cellular nanoparticle decrease as result of exocytosis and cell division (“nanoparticle dilution”). *n* = 3 independent experiments; *MFI* median fluorescence intensity of the cell population; *asterisks* indicate significant differences (*P* ≤ 0.05) to the initial value (“0 h” follow-up; 100 %), *different letters* indicate significant differences (*P* ≤ 0.05) between different time points. **b** The occurrence of cerium (Ce) in the supernatant of endothelial cells, which were previously exposed to CeO_2_ nanoparticles and which were followed up after washing and cell culture medium exchange (nanoparticle free medium), revealed the occurrence of exocytosis of intracellular nanoparticles. The lower Ce supernatant concentrations which were found with increasing follow-up time (48 and 72-h follow-up time) in comparison to 24-h follow-up time indicate a re-uptake of exocytosed nanoparticles in cells. The Ce content in supernatants of cells, which were not treated with nanoparticles, was below the detection limit. *n* = 2 independent experiments

The analysis of the ability of HMEC-1 to exocytose nanoparticles generally showed a continuing reduction in the MFI of the cell population with increasing follow-up time after the cells were treated with nanoparticles (Fig. 2a). For example, cells which were exposed to nanoparticles for 24 h (correspond to follow-up time point “0 h”) and then washed and supplied with fresh nanoparticle free medium, revealed approximately 70 % of the initial MFI value (value at “0 h” follow-up) at 48 h after the medium exchange (“48 h” follow-up time), 35 % after 72 h, only 1.7 % after 120 h, and 0.3 % after 240 h (Fig. 2a). Since the fluorescence correlated with the internalized nanoparticle amount, this decrease indicated a nanoparticle reduction within the cell population.

The Ce content in the cell culture medium supernatants of cells, which were exposed to CeO_2_ nanoparticles for 24 h and then processed as mentioned above, confirmed the exocytosis of intracellular nanoparticles at the different time points (Fig. 2b). The cell culture supernatants of the 48 and 72-h follow-up presented lower Ce concentrations than the 24 h follow-up samples (Fig. 2b). These findings could be a result of partial re-uptake of already exocytosed nanoparticles in HMEC-1.

#### Intracellular re-arrangement of nanoparticles with increasing time after exposure and role of cellular constituents in nanoparticle exocytosis

**Fig. 3:**
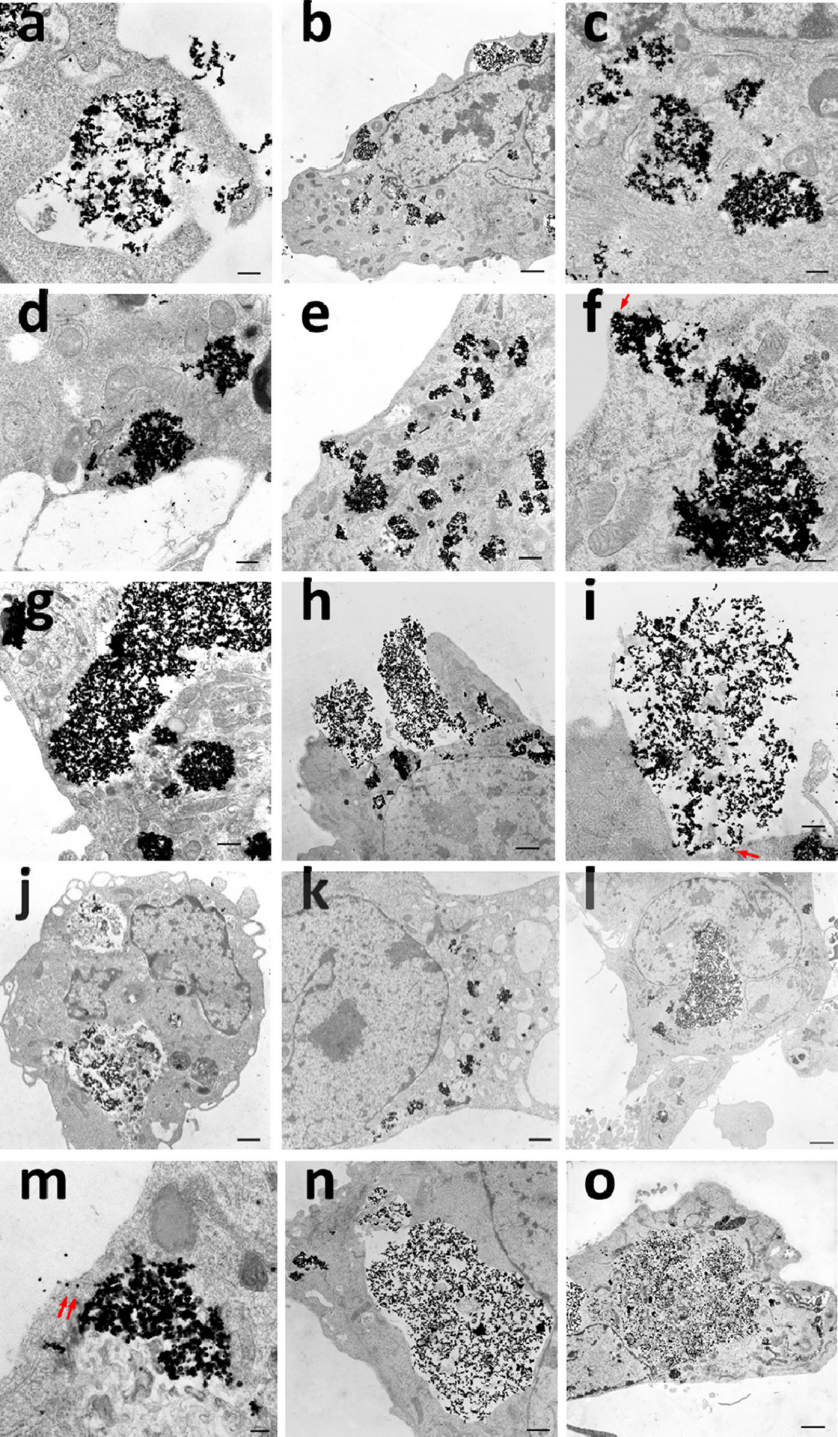
Intracellular localization of nanoparticles with increasing time after exposure and after inhibitor application. TEM images of HEMC-1 after treatment with 10 µg/ml CeO_2_ nanoparticles for 24 h show endocytosis (**a**) and the internalized nanoparticles in endosomes (**b**) as well as in the cytosol (**c**). TEM images of the localization of the nanoparticles 24 h after washing and medium exchange with nanoparticle free medium (**d**–**i**) revealed clearly the initiation (**e**, **f**) and occurrence (**h**, **i**) of exocytosis of the internalized nanoparticles. Cells treated with brefeldin A (**j**, **k**; 0.1 µg/ml, 24 h), nocodazole (**l**, **m**; 10 µg/ml, 24 h), or MβcD (**n**, **o**; 10 mM, 2 h) revealed no or hardly any exocytosis (**m**), but the localization of nanoparticles in large endosomes (**j**, **l**, **n**) or in the cytoplasm (**k**, **m**, **o**). The occurrence of nanoparticles in the cytoplasm indicated endosomal perforation. *Arrows* (**f**, **i**, **m**) point to the cytosolic nanoparticles which are shortly before exocytosis. *Scale bars*: 0.1 (**m**); 0.3 µm (**b**, **c**, **d**, **f**); 0.5 µm (**i**); 1.0 µm (**e**, **g**, **j**, **k**, **n**); 1.5 µm (**a**, **h**); 2.0 (**l**, **o**)

The investigation of the intracellular localization of nanoparticles with increasing follow-up time after exposure revealed a re-arrangement of the nanoparticles within cells. TEM images of the follow-up time point “0 h” (HMEC-1 were treated with CeO_2_ nanoparticles for 24 h) showed the endocytosis process of the nanoparticles (Fig. 3a). The internalized nanoparticles were found mainly in endosomes (Fig. 3b) and partly in the cytosol (Fig. 3c). At this time point no exocytosis or exocytosis initiation could be observed.

TEM images of HMEC-1, which were firstly treated with nanoparticles for 24 h and then washed and supplied with fresh nanoparticle free medium, (Fig. 3d–i) disclosed 24 h after the medium exchange (“24 h” follow-up time) a localization of the nanoparticles partly in the cytosol (Fig. 3d) and rarely in lysosomes (Fig. 3e) as well as a migration of the endosomal vesicles with the nanoparticles toward the plasma membrane (Fig. 3e–h). Moreover, the initiation (Fig. 3e, f) and occurrence (Fig. 3h, i) of exocytosis of the internalized nanoparticles were seen clearly. Since hardly no extracellular nanoparticles were observed in TEM images of cells which have been treated with inhibitor (Fig. 3j–l, n, o), the extracellular localization of nanoparticles in relation to non-inhibited cells (Fig. 3h, i) evidences the presence of exocytosis. The exocytosis process seems to occur mainly by fusion of the endosomal membrane with the plasma membrane.

Cells, which were treated after nanoparticle exposure with the inhibitors brefeldin A (Fig. 3j, k; 0.1 µg/ml, 24 h), nocodazole (Fig. 3l, m; 10 µg/ml, 24 h), or MβcD (Fig. 3n, o; 10 mM, 2 h) to detect the role of cellular constituents in the exocytosis of nanoparticles, presented considerably large nanoparticle-containing endosomes (Fig. 3j, l, n). This indicated the inhibition of exocytosis. Occasionally, a localization of nanoparticles in secondary lysosomes was detected for brefeldin A and MβcD-treated cells. In analogy to the native cells (no inhibitor, Fig. 3c), nanoparticles were also found in the cytoplasm of cells additionally exposed to inhibitors (Fig. 3k, m, o). In both cases, the occurrence of nanoparticles in the cytoplasm indicated the presence of endosomal perforation possibly due to a too high amount of nanoparticles which accumulated within the endosomes. Interestingly, it seems that nanoparticles which already translocated to the cytoplasm could be expelled from endothelial cells due to the close vicinity of cytosolic nanoparticles to the plasma membrane in representative TEM images (Fig. 3f, i, m (arrows)). TEM images of nocodazole-treated cells showed a migration of small endosomes containing nanoparticles toward the plasma membrane. It was detected that nocodazole permitted an occasional exocytosis of some single nanoparticles (Fig. 3m), which was hardly the case for brefeldin A or MβcD. Overall, cells treated with one of the three inhibitors showed no or hardly any exocytosis whereby MβcD showed the best inhibitory effect among all the applied inhibitors.

**Fig. 4:**
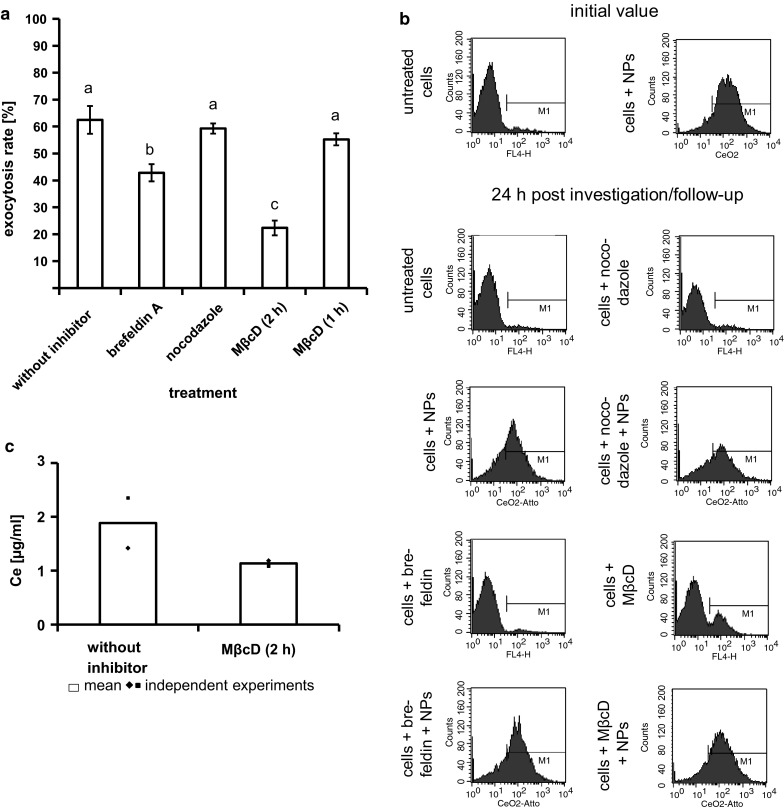
Strong inhibitory effect of MβcD and brefeldin A indicates the important role of plasma membrane cholesterol and Golgi-to-cell-surface-transport, respectively, during nanoparticles exocytosis. **a** The average exocytosis rate of CeO_2_ nanoparticles (treatment dose 1 µg/ml for 24 h) within 24 h was 62 ± 5 %. Nocodazole led to no obvious inhibition of nanoparticle exocytosis (exocytosis rate: 59 ± 2 %). The highest inhibition of exocytosis was caused by MβcD with an exposure time of 2 h indicating an important role of plasma membrane cholesterol for exocytosis. Brefeldin A treatment resulted also in an inhibition of exocytosis revealing an involvement of Golgi-to-cell-surface-transport in exocytosis process. *Different letters* indicate significant differences (*P* ≤ 0.05) between the various treatments. *n* ≥ 3 independent experiments; **b** Histograms of a representative flow cytometry analysis; NPs: nanoparticles. **c** The determination of cerium (Ce) in the supernatant of HMEC-1, which were exposed to nanoparticles for 24 h, revealed 24 h after washing and cell culture medium exchange (nanoparticle free medium) a higher amount of Ce than the supernatants of HMEC-1 which were additionally treated with MβcD-containing cell culture medium after washing and medium exchange. This confirmed the inhibition of exocytosis by MβcD. The Ce content in supernatants of cells, which were not treated with nanoparticles, was below the detection limit. *n* = 2 independent experiments

To quantify the inhibitory effect of each inhibitor, the assessment of the exocytosis rate via flow cytometry analysis was performed (Fig. 4a, b) as described in the experimental section. The optimal nanoparticle concentration for the flow cytometry analysis was 1 µg/ml (exposure time: 24 h) as it was demonstrated in a preliminary test (data not shown). Within 24 h after medium exchange, an average exocytosis rate of 62 ± 5 % was detected (Fig. 4a). In comparison to the other inhibitors, MβcD with an exposure time of 2 h led to the strongest inhibition of nanoparticle exocytosis (exocytosis rate: 22 ± 3 %, Fig. 4a). MβcD with a shorter exposure time (1 h) led to a weaker inhibition of exocytosis (exocytosis rate: 55 ± 2 %, Fig. 4a) indicating that the cholesterol content of cell membranes played an important role in the exocytosis process.

As a consequence of brefeldin A treatment, an exocytosis rate of 43 ± 3 % was found, which means that an inhibition of nanoparticle exocytosis occurred (Fig. 4a). The applied inhibitor nocodazole caused no obvious inhibition of nanoparticle exocytosis (exocytosis rate: 59 ± 2 %, Fig. 4a). Thus, the transfer from distal Golgi compartments to the cell surface influenced the exocytosis process of the CeO_2_ nanoparticles to a higher extent than the microtubule-associated transport.

The detected Ce concentration in cell culture medium supernatants of cells, which were washed and supplied with fresh nanoparticle free cell culture medium after nanoparticle exposure, in comparison to those of cells, which were additionally treated with the inhibitor MβcD, confirmed the inhibitory effect of MβcD in the exocytosis of CeO_2_ nanoparticles (Fig. 4c).

## Discussion

Our study has yielded the following results: (1) Endothelial cells are able to release CeO_2_ nanoparticles via exocytosis to reduce the intracellular nanoparticle accumulation. (2) A partly re-uptake of the already released nanoparticles occurs. (3) After uptake the nanoparticles were mainly localized in endosomes, which migrated toward the plasma membrane and released the nanoparticles in the extracellular environment via membrane fusion. The nanoparticles were partly found in the cytosol and rarely in lysosomes. Apart from their release via fusion of vesicles with the plasma membrane, nanoparticles seem to also be able to directly leave the cells. (4) The cholesterol content of the cell membrane plays an important role in the exocytosis process. The transfer from distal Golgi compartments to the cell surface influenced the exocytosis process of the CeO_2_ nanoparticles more than the microtubule-associated transport.

In the present study, flow cytometry analysis showed a reduction of MFI in the cell population with increasing follow-up time after extracellular nanoparticle exposure. This was attributed, at least in part, to exocytosis. However, considering the doubling time of the used HMEC-1 cells (approximately 33.6 h), it should be taken into account that the MFI decrease of flow cytometry analysis, besides exocytosis, may result in part also from cell division which can lead to a nanoparticle dilution within the cell population as it was shown by other researches (Errington et al. 2010; Kim et al. 2012; Summers et al. 2011). It is discussed that the distribution of nanoparticles during cell division occurs asymmetrically (Errington et al. 2010; Summers et al. 2011) with the aim to inherit the foreign substance mainly by one of the daughter cells in order to ensure survival of the remaining cell population (Summers 2010). This means that the cell population contained cells with a high nanoparticle amount on one side, and on the other cells with low or even no nanoparticles. On account of the influence of cell division on the MFI, it is necessary to use not only flow cytometry analysis for exocytosis studies, but also complementary methods to verify the obtained results. Thus, in this study the exocytosis process was confirmed by TEM, flow cytometry, and ICP-MS analysis in combination with certain inhibitors.

In this context, the occurrence of Ce in cell culture medium supernatants of cells after several time points after finalization of the nanoparticle exposure confirmed the exocytosis of CeO_2_ nanoparticles from endothelial cells. It was perceived that with longer follow-up time (48 h; 72 h) the Ce concentrations of the corresponding cell culture supernatants were lower than those related to a 24-h follow-up time. This finding can be explained by a re-uptake of already exocytosed nanoparticles by HMEC-1, because endocytosis and exocytosis seem to be dynamic processes which occur simultaneously and are dependent from the nanoparticle amount outside and inside of cells (Chu et al. 2011).

TEM analysis indicated that the exocytosis process occurred mainly via fusion of vesicular (especially endosomal) membranes with the plasma membrane, leading to the release of the vesicular content into the extracellular environment. But also a further exocytosis way was detected: The rupture of endosomes which contained nanoparticles (perhaps due to the high amount of internalized nanoparticles) led to the translocation of released nanoparticles to the cytoplasm and from there the nanoparticles seemed to be expelled directly from endothelial cells presumably via unspecific mechanisms.

The application of the different inhibitors clarified, which intracellular pathways are important for exocytosis. In this context, after treatment of cells with MβcD a strong inhibitory effect on exocytosis was observed via TEM (very large endosomes of cells with MβcD treatment and hardly no fusion of vesicles containing nanoparticles with the plasma membrane), flow cytometry (lower exocytosis rate of MβcD-treated cells in comparison to non-treated cells), and ICP-MS analysis (lower Ce concentrations in cell culture medium supernatants of cells treated with MβcD in comparison to non-treated cells). MβcD, a cyclic oligosaccharide, consists of 7 glycopyranose units, which form a hydrophobic cavity, where cholesterol will be incorporated (Pitha et al. 1988) and making it soluble in the aqueous cell culture medium (Klein et al. 1995; Ohtani et al. 1989). MβcD removes the cholesterol selectively from the cell plasma membrane without membrane incorporation (Klein et al. 1995; Ohtani et al. 1989). Thus, the inhibitory effect of MβcD on exocytosis of CeO_2_ nanoparticles from endothelial cells indicated a significant role of cholesterol for nanoparticle exocytosis. A shorter incubation time with MβcD resulted in a much lower inhibition of exocytosis, most likely due to an insufficient reduction in the cell membrane cholesterol content. This finding emphasizes the importance of cell membrane cholesterol for exocytosis processes. Interestingly, the detected inhibitory effect of cholesterol depletion on CeO_2_ nanoparticle exocytosis from endothelial cells is concordant with findings related to maltodextrin nanoparticles (*ø* ≈ 60 nm by laser light scattering) exposed to airway epithelium cells (Dombu et al. 2010), but in contrast to published data regarding polymer nanoparticles (*ø* ≈ 80 nm by DLS) and MDCK (He et al. 2013a) or Caco-2 epithelial cells (He et al. 2013b), where the extraction of cholesterol improved exocytosis. This indicates a nanoparticle and/or cell type dependency of nanoparticle exocytosis pathways. A study comparing the ability of three different cell types (lung carcinoma (A549), bronchial epithelial (16HBE), and primary adult stem cells (MSC)) to exclude gold nanorods within 72 h from the cells reported that only the stem cells excreted the internalized nanoparticles (Wang et al. 2011), which also suggests a cell type dependency in nanoparticle exocytosis. Cell type-specific differences [human esophageal epithelial cells (NE083) and human lung carcinoma cells (H1299)] were also observed in the exocytosis of silica nanoparticles (Chu et al. 2011).

Brefeldin A treatment also caused an inhibitory effect on nanoparticle exocytosis, but to a lesser extent than MβcD. TEM images of nocodazole exposed cells revealed an occasional exocytosis of some single nanoparticles, but overall this was very low in comparison to the exocytosis process of non-treated cells. The contradictory findings between flow cytometry and TEM analysis in relation to nocodazole co-incubation of cells (flow cytometry: ineffectiveness of inhibition, quantitative analysis; TEM: only rarely exocytosis; snap-shot analysis) emphasize the importance to verify the exocytosis results with more than one analysis method. Overall, the results regarding brefeldin A and nocodazole suggest that the transfer from distal Golgi compartments to the cell surface [should be inhibited by brefeldin A (Miller et al. 1992)] influences the exocytosis process of the CeO_2_ nanoparticles more than the microtubule-associated transport [should be inhibited by nocodazole (Peterson and Mitchison 2002)]. The involvement of Golgi to plasma membrane pathway in nanoparticle exocytosis seems to be generally of importance as this pathway was also shown for other nanoparticles (polymer nanoparticles, *ø* ≈ 80 nm by DLS) and cell systems [MDCK and Caco-2 epithelial cells (He et al. 2013a, b)].

On the whole, the present study showed that endothelial cells are able to excrete internalized nanoparticles to control their nanoparticle loading mainly via the plasma membrane cholesterol-dependent mechanisms. While the Golgi to plasma membrane pathway is also important for CeO_2_ nanoparticle exocytosis, the microtubule-associated transport seems to play only a marginal role. The exocytosis of nanoparticles should be very important for the cell to prevent cell damage with the final consequence of cell death as a result of excessive nanoparticle enrichment.

## Conclusions

It can be concluded that endothelial cells, which are the first barrier after nanoparticles arrived at the blood system, are able to remove internalized nanoparticles by exocytosis processes. After uptake, the internalized nanoparticles are re-arranged within cells—the nanoparticle containing vesicles (mainly endosomes) migrates to the plasma membrane. The exocytosis process occurs mainly by fusion of endosomes with the plasma membrane, but probably also—to a less extent—by a direct release of free cytosolic nanoparticles. The already exocytosed nanoparticles can also be re-taken up by cells. An important role of plasma membrane cholesterol was identified for the exocytosis process. Furthermore, the transfer from distal Golgi compartments to the cell surface seems to influence the exocytosis process of the CeO_2_ nanoparticles more than the microtubule-associated transport. A sufficient exocytosis of nanoparticles should protect endothelial cells for adverse effects of nanoparticle accumulation.
